# Editorial: Physiological alterations of nematodes influenced by cross-phylum symbioses

**DOI:** 10.3389/fphys.2024.1417354

**Published:** 2024-05-08

**Authors:** Michael S. Werner, Nathan Schroeder

**Affiliations:** ^1^ School of Biological Sciences, The University of Utah, Salt Lake City, UT, United States; ^2^ Department of Crop Sciences, University of Illinois at Urbana-Champaign, Champaign, IL, United States

**Keywords:** nematode, symbiosis, entomopathogen, vivipary, dauer formation

Nematodes are among the most abundant and widely distributed animal phyla. It should, therefore, be unsurprising that a wide range of physiological adaptations have emerged in this group. Cross species interactions are known to directly impact evolutionary trajectories. In this Research Topic, authors have shared their findings on diverse adaptations of diverse nematode species.

The genus *Bursaphelenchus* comprises multiple species that associate with beetles as a morphologically distinct developmental stage called dauer. The pine-wilt nematode *B. xylophilus*, which causes extensive damage to pine trees in Asia and Europe, has evolved two separate dauer stages (Tanaka et al., 2017). A DIII dauer stage forms under conditions of low food availability, while a DIV dauer is induced by the presence of a beetle vector. In contrast, the related *B. okinawensis* only forms a single DIII stage. Using a series of laboratory manipulations, Kirino et al., examined the factors regulating dauer entry in *B. okinawensis*. Like *C. elegans* and many other species, dauer formation of *B. okinawensis* was induced under conditions of crowding and elevated temperature. However, exposure to pupae of Monochamus beetles also induced dauer formation in *B. okinawensis*. Interestingly, despite being induced by exposure to Monochamus, only a small fraction of dauers used the beetle as a vector suggesting distinct life-history strategies for these two species of *Bursaphelenchus*.

Yamashita et al. investigated a bacterial feeding nematode, *Tokorhabditis tufae*, which is adapted to extreme levels of heavy metal in Mono Lake, California. Additionally, this species is one of a handful of nematode species suggested to reproduce through vivipary (i.e., giving birth to live young) (Kanzaki et al., 2021). Many nematodes display a form of facultative vivipary known as *endotokia matricida* where the young hatch within the mother and eat their way out. However, very few nematode species are proposed to lay their offspring as hatched larvae. Yamashita et al. show that *T. tufae* embryos are more permeable to fluorescent dyes, have a greatly reduced eggshell lacking several layers found in other bacterial feeding nematode species and grow in size during development. These findings are highly suggestive of true vivipary. It will be interesting to know the ecological factors that have led to the evolution of this unusual developmental adaptation.

Also in this Research Topic, Tarasco et al. write a comprehensive review of entomopathogenic nematodes (EPN) with a particular focus on their use as biocontrol agents against insect crop pests. The authors describe in detail several of the methods used for sampling and extraction, and modern methods for molecular identification of EPNs and their bacterial symbionts. We anticipate this will be a highly useful resource for experienced members of the community and a thorough but accessible introduction for newcomers.

In Lefoulon et al. the authors investigated the cost of mutualistic relationships between EPNs and their bacterial symbionts. Entomopathogenic nematodes serve as vectors to deliver bacteria to insect hosts. When inside the body cavity the bacteria are released, replicate, and often cause mortality within 48 h. Meanwhile, the nematodes can feed on the expanding population of bacteria and the decaying insect. Once the cadaver is depleted of nutrients, nematodes leave as infective juveniles (IJs), carrying the pathogenic bacteria with them in search of their next host. One of the most powerful systems to understand this fascinating tripartite symbiosis is the nematode genus *Steinernema*, it’s bacterial symbionts *Xenorhabdus*, and insect larvae from a wide range of species (although commonly the greater wax moth *Galleria mellonella*). While there are clear benefits of *Steinernema* to carry *Xenorhabdus*, there are intriguing data points that suggest tradeoffs may also exist.

First, while most *Steinernema* species are dependent on their bacterial symbionts for reproduction, some are not (Sicard et al., 2003), suggesting that there are degrees of symbiosis. The different degrees likely reflect evolutionary trade-offs between dependency of *Steinernema* on *Xenorhabdus* vs. other bacteria. Second, and more directly, *Steinernema carpocapsae* IJs have greater longevity without their bacterial symbionts at 25°C and 30°C (Mitani et al., 2004) and the abundance of their symbionts negatively correlates with IJ lifespan (Emelianoff et al., 2008). To investigate the molecular basis of these tradeoffs, Lefoulon et al. performed RNA-seq of two *Steinernema* species with or without their cognate bacterial symbionts, both *in vivo* (with an insect host) or *in vitro*. Their analysis revealed several shared gene expression patterns and gene-ontology/KEGG pathways across conditions and species—as well notable differences. The manuscript thoroughly describes each comparison, and the data will undoubtedly be a valuable resource.

The authors primarily focused on the preponderance of metabolic genes that were differentially expressed between *in vivo* and *in vitro* conditions, which support previous observations (Hatab et al., 1998) and likely reflect lower levels of available nutrients in the absence of the insect host. It was particularly interesting that downregulation of trehalose metabolism was exaggerated in aposymbiotic IJs. There were also interesting trends in stress response and mTOR signaling pathway genes between aposymbiotic IJs and *Xenorhabdus*-colonized conditions. Finally, there were several differentially expressed genes implicated in venom production in the presence or absence of *Xenorhabdus*.

These shifts are attractive candidates for identifying trade-offs of bacterial symbionts with their nematode hosts, and some like the metabolic and stress response pathways may link back to previous observations that *Xenorhabdus* negatively affects the longevity of IJs. However, ultimately it will be important to perform functional experiments to test these hypotheses. Excitingly, a hermaphroditic *Steinernema* species (*Steinernema hermaphroditum*) has recently been developed as a model organism to do exactly that (Cao et al., 2021). The future of understanding nematode:bacterial symbioses, unlike the host insect’s, looks bright.
